# Short- and Long-Term Social Recognition Memory Are Differentially Modulated by Neuronal Histamine

**DOI:** 10.3390/biom11040555

**Published:** 2021-04-09

**Authors:** Barbara Rani, Bruna Silva-Marques, Rob Leurs, Maria Beatrice Passani, Patrizio Blandina, Gustavo Provensi

**Affiliations:** 1 Department of Health Sciences (DSS), Section of Clinical Pharmacology and Oncology, University of Florence, 50139 Florence, Italy; barbara.rani@unifi.it (B.R.); beatrice.passani@unifi.it (M.B.P.); 2 Department of Physiotherapy, Center of Biological Sciences and Health, Federal University of Sao Carlos, São Carlos-SP 13565-905, Brazil; silvamarquesufscar@gmail.com; 3 Department of Neurosciences, Psychology, Drug Research and Child Health (NEUROFARBA), Section of Pharmacology of Toxicology, University of Florence, 50019 Florence, Italy; patrizio.blandina@unifi.it; 4 Amsterdam Institute for Molecules, Medicines and Systems (AIMMS), Division of Medicinal Chemistry, Faculty of Science, Vrije Universiteit Amsterdam, 1081 HZ Amsterdam, The Netherlands; r.leurs@vu.nl

**Keywords:** social discrimination, memory, histamine, acetylcholine, H_3_R, histidine decarboxylase

## Abstract

The ability of recognizing familiar conspecifics is essential for many forms of social interaction including reproduction, establishment of dominance hierarchies, and pair bond formation in monogamous species. Many hormones and neurotransmitters have been suggested to play key roles in social discrimination. Here we demonstrate that disruption or potentiation of histaminergic neurotransmission differentially affects short (STM) and long-term (LTM) social recognition memory. Impairments of LTM, but not STM, were observed in histamine-deprived animals, either chronically (*Hdc^−/−^* mice lacking the histamine-synthesizing enzyme histidine decarboxylase) or acutely (mice treated with the HDC irreversible inhibitor α-fluoromethylhistidine). On the contrary, restriction of histamine release induced by stimulation of the H_3_R agonist (VUF16839) impaired both STM and LTM. H_3_R agonism-induced amnesic effect was prevented by pre-treatment with donepezil, an acetylcholinesterase inhibitor. The blockade of the H_3_R with ciproxifan, which in turn augmented histamine release, resulted in a procognitive effect. In keeping with this hypothesis, the procognitive effect of ciproxifan was absent in both *Hdc^−/−^* and αFMH-treated mice. Our results suggest that brain histamine is essential for the consolidation of LTM but not STM in the social recognition test. STM impairments observed after H_3_R stimulation are probably related to their function as heteroreceptors on cholinergic neurons.

## 1. Introduction

The term social memory encompasses two different cognitive processes. The first, social learning, refers to the ability to learn from others. The second, social recognition, is defined as the ability to recognize a familiar or novel conspecific [1]. These processes are of paramount importance for social species since the accurate recognition of a specific individual associated with the interpretation of specific environmental cues lead to the unfolding of the appropriate behavioral response such as: approach, investigation, attack or mounting [2]. All these complex, either cooperative or competitive, behaviors have been selected and persisted during the evolution due to their key roles for social hierarchy, mate and offspring recognition, territorial defense, interspecies recognition, and for the general establishment and maintenance of groups [3].

In rodents, social recognition memory can be evaluated by using their instinctive tendency to investigate unfamiliar conspecifics more persistently than familiar ones. Different experimental designs can be used: in the habituation/dishabituation paradigm an adult animal is exposed to an unfamiliar, juvenile subject. Subsequently, the adult is re-exposed to the same animal in different experimental sessions. A decrease in social investigation upon repeated encounters is interpreted as an index for social recognition [4,5,6]. An alternative paradigm is the social discrimination test. This task shares the initial phase with the habituation/dishabituation paradigm. However, during the retention test, the familiar juvenile and a novel conspecific are simultaneously presented to the adult animal. Due to their innate tendency to investigate novelty, preferential exploration of the novel juvenile is expected [7]. The establishment of the correct identity of an individual depends on different sensory cues. Humans and other primates use auditory and visual information for coding information pertaining to the physical characteristics of an individual. For other animals, including rodents, chemosensory cues in the form of olfactory or pheromonal signals are more relied upon to encode social information [3,8,9]. As for other types of memory, the acquired sensory social information goes through the process of consolidation, in order to stabilize the labile short-term memory (STM) to ensure long-term storage [10,11]. Such a process involves a wide and complex circuitry, which includes the dorsal and ventral hippocampus, basolateral and medial amygdala, insular cortex, lateral septum and the medial prefrontal cortex [6,12,13] in which different hormones such as oxytocin and vasopressin [14,15] and neurotransmitters like acetylcholine [16], dopamine [17] noradrenaline [18] and histamine [19] play key modulatory roles.

In the brain, the cell bodies of all histaminergic neurons are in the tuberomamillary nucleus of the hypothalamus [20,21]. These cells project to multiple brain areas such as the septum/diagonal band nucleus, the dorsal hippocampus, the basolateral amygdala and the cortex [22,23], all regions critically involved in memory processing [24,25]. Accordingly, there is extensive evidence indicating that histamine acts as a major regulator of consolidation and retrieval of different types of memory, including social recognition as reviewed in [19,26,27]. It was previously reported that infusion of histamine or its precursor histidine into the rat lateral ventricles improved whereas the blockade of histamine biosynthesis impaired social recognition learning [28]. Similar results were obtained when brain histamine levels were modulated following the injection of H_3_R ligands: treatment with the H_3_R antagonist thioperamide, which increases brain histamine availability, facilitates social recognition. Conversely, reduction of neuronal histamine release following injection of immepip, an H_3_R agonist, elicited amnesic effects [28]. Several studies using different H_3_R antagonists confirmed and expanded these observations [29,30,31,32,33,34]. However, these studies were performed using the habituation/dishabituation paradigm in rats using short inter trial intervals (40–120 min), therefore they were not suitable to explore putative differential effects on short- and long-term memories. Moreover, many of the previous studies were focused on H_3_R antagonists given before or shortly after training, demonstrating the drugs’ effects on memory acquisition and/or consolidation, but not on retrieval. Thus, in the present study we used a combination of genetic and pharmacological approaches to study the involvement and temporal dynamics of the requirement of the brain histaminergic system on social recognition learning using the social discrimination paradigm in mice.

## 2. Materials and Methods

### 2.1. Animals

Genetically modified mice carrying a histidine decarboxylase gene deletion (Hdc^−/−^) as well as wild type (Hdc^+/+^) mice were used in this study. The Hdc^−/−^ animals are descendent from the mouse strain generated by Ohtsu et al., 2001 [35] and were backcrossed to C57Bl/6J strain for 10 generations at the animal facility of the Centro di Servizi per la Stabulazione di Animali da Laboratorio (CeSAL, Florence, Italy) of the University of Florence. Animals were housed in humidity and temperature-controlled rooms (22–24 °C) allowed free access to food (4RF21; Mucedola s.r.l., Settimo Milanese, Italy) and water, and kept on a 12-h light/dark cycle (lights start at 7:00 a.m.). Breeding, housing, and all the experimental procedures were conducted in accordance with the Council Directive of the European Community (2010/63/EU) and the Italian Decreto Legislativo 26 (13 March 2014), approved by the Animal Care Committee of the University of Florence and Italian Ministry of Health and supervised by a veterinarian. Every effort was made to minimize animal suffering and to reduce the number of animals used.

All mice were genotyped by PCR amplification before the experiments following a previously described protocol [36,37]. Juvenile (4–5 weeks old) and adult (8–9 weeks old) mice were used in the experiments. Male mice were used to decrease within-group variability due to hormonal fluctuations during estrous cycle in female mice. Animals were handled for at least 4 days before experiments begun, to let them acclimatize to human contact. All the experiments were conducted between 9:00 a.m. and 4:00 p.m.

### 2.2. Compounds

VUF16839 was synthesized by the group of Prof. Rob Leurs at Vrije Universiteit Amsterdam as previously described [38]. Ciproxifan maleate was provided by Prof. Holger Stark (Heinrich-Heine-Universität Düsseldorf, Düsseldorf, Germany).). Donepezil hydrochloride was purchased from Sigma-Aldrich (Milan, Italy). These drugs were freshly prepared and dissolved in physiological saline (0.9% wt/vol. NaCl) to permit the systemic injection (i.p.) of a constant volume of 1 mL/100 g body weight to each mouse. α-fluoromethylhistidine (αFMH) synthesized at Johnson & Johnson Laboratories was a kind gift of Dr. Nicholas Carruthers. αFMH was dissolved in physiological saline to obtain a solution with a final concentration of 1 µg/µL and infused directly into the lateral ventricles as described below.

### 2.3. Stereotaxic Surgery and i.c.v. Infusion Procedure

Mice were anesthetized using a mixture of ketamine (15 mg/kg) and xylazine (2.5 mg/kg) and placed in a stereotaxic frame equipped with a mouse adapter and ear bars (Kopf Instruments, Tujunga, CA, USA). A stainless-steel cannula (7 mm in length, outer diameter 0.5 mm, and inner diameter 0.25 mm) was then implanted in the lateral ventricle and fixed to the skull using dental cement. The following coordinates were used according to the mouse brain atlas [39]: antero-posterior (AP) −0.3 mm; lateral (L) +1 mm; dorsoventral (DV) −1 mm. Animals were then left to recover for a period of 7 days. In the infusion day a stainless-steel injection micro-needle (outer diameter 0.25 mm) was connected through a polyethylene catheter to a 1000 µL Hamilton precision syringe and then lowered into the lateral cerebral ventricle (DV 2.4 mm). In total, 5 µL of a solution containing αFMH (1 µg/µL in sterile saline) were delivered using an infusion pump at 1 µL/min flow. Animals in the control group received equal volumes of sterile saline. The needle was left in place for one additional min after each infusion [40,41].

### 2.4. Social Discrimination Test

Adult male mice (8–9 weeks old) behavior was assessed in an open-field Plexiglas arena (45 × 25 cm and 20 cm high) placed in a sound attenuated room. The assay paradigm comprises three sessions: habituation, training, and test. In the first session, mice were placed in the arena containing two empty pencil-wire cups positioned on opposing sides and left free to explore for 10 min. Then, 24 h after this session, a juvenile mouse (stimulus, 3−4 weeks old), which had no prior contact with the subject mice, was placed under one of the wire cups while the other cup remained empty. The subject mouse was then placed in the arena and was left free to explore it for 10 min. During the third session, performed after different intervals, the same stimulus animal was again placed under the wire cup and a novel unfamiliar juvenile mouse was placed under the opposing cup. Experimental mice were then placed again in the arena and tested for discrimination between novel and familiar mouse in a 10 min session. Each experimental mouse was subjected to the procedure separately, and care was taken to remove any olfactory/taste cues by cleaning carefully the arena and wire cups between trials. The positions of the social stimuli (empty × social; familiar × novel) were counterbalanced across subjects and trials to prevent bias due to place preference. All juvenile stimulus mice were habituated to remain under the wire cups for 30 min during several days before behavioral testing. The animal’s behavior during all sessions was videotaped, and the time spent actively exploring the stimuli was analyzed by experienced observers unaware of the experimental groups. Exploration was defined as direct snout-to-cup contact, and the time spent climbing on the cups was not considered. Data are expressed as a percentage of time spent exploring each cup (social × nonsocial during the second session or familiar × novel during the third session). The raw exploration time data were also employed to determine social and discrimination indexes, according to the following equations:

$$\mathrm{Sociability}\;\mathrm{Index}\;\left(\mathrm{SI}\right)=\;\frac{\mathrm{time}\;\mathrm{exploring}\;\mathrm{social}\;\left(\mathrm{tS}\right)-\;\mathrm{time}\;\mathrm{exploring}\;\mathrm{non}\;\mathrm{social}\;\left(\mathrm{tNS}\right)}{\mathrm{total}\;\mathrm{exploration}\;\mathrm{time}\;\left(\mathrm{tS}+\mathrm{tNS}\right)}$$

$$\mathrm{Discrimination}\;\mathrm{Index}\;\left(\mathrm{DI}\right)=\;\frac{\mathrm{time}\;\mathrm{esploring}\;\mathrm{novel}\;\left(\mathrm{tN}\right)-\;\mathrm{time}\;\mathrm{exploring}\;\mathrm{familiar}\;\left(\mathrm{tF}\right)}{\mathrm{total}\;\mathrm{exploration}\;\mathrm{time}\;\left(\mathrm{tN}+\mathrm{tF}\right)}$$

### 2.5. Statistical Analysis

Data from behavioral experiments presented in graphs are expressed as mean and ± S.E.M and were analyzed using Graphpad Software (version 6.0, San Diego, CA, USA). The percentage of time the animals spent exploring the different stimuli was analyzed with two-way Analysis of Variance (ANOVA), whereas the sociability and discrimination indexes were analyzed with unpaired *t*-test or two-way ANOVA as appropriate for the experimental design. The source of the detected significances was determined by Bonferroni’s multiple comparison post-hoc test. *p* values less than 0.05 were considered statistically significant. The number of mice per experimental group is indicated in their respective figure legends. Details of the statistical analysis including critical values are described in the Supplementary Tables S1–S4.

## 3. Results

### 3.1. Neither Genetic Manipulation nor Pharmacological Interventions Affected Mice Sociability

**Figure 1:**
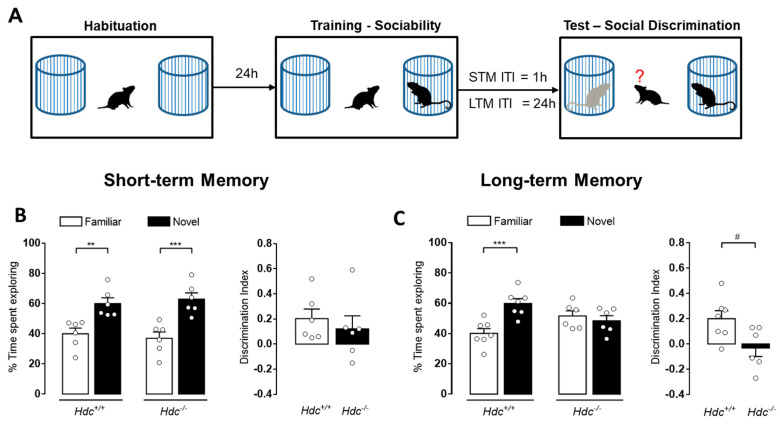
Impact of chronic histamine depletion on short- and long-term social recognition memory. (**A**) Schematic drawing showing the sequence of the behavioral procedures. Hdc^+/+^ and Hdc^−/−^ male mice were submitted to the retention test 1 h (**B**) or 24 h (**C**) after training. Shown are means ± S.E.M. of 6–7 animals per experimental group. *** *p* < 0.001, ** *p* < 0.01 by two-way ANOVA and Bonferroni’s MCT; ^#^ *p* < 0.05 by unpaired *t*-test.

In this work we decided to use the social discrimination paradigm because the experimental design allows the evaluation of two critical but distinguishable aspects of social behavior: social preference or sociability, as well as social recognition memory. By evaluating the performance of the experimental animals during the training phase (Figure 1A), it is possible to determine their social motivation, defined as the natural propensity to spend time exploring a social stimulus compared to the time spent alone exploring the empty cylinder. Deficits on sociability may influence animals’ preference for social novelty evaluated in the retention test session and defined as propensity to spend time with a previously unencountered mouse rather than with a familiar mouse [42]. Moreover, social preference deficits have been extensively described in several Autism Spectrum Disorder mouse models [43]. As shown in Table 1 all the animals used in the different experiments spent significantly more time exploring the cup containing a sex-matched conspecific juvenile, as revealed by the positive sociability index values. Remarkably, neither the disruption nor the potentiation of the histaminergic transmission altered natural social preference, suggesting that neuronal histamine does not play a key role on mice sociability.

### 3.2. Blockade of Histamine Synthesis Impairs Long- but Not Short-Term Social Recognition Memory

To investigate short-term memory (STM), during the retention test performed 1 h after training, normal (Hdc^+/+^) and chronically histamine deprived (Hdc^−/−^) mice were simultaneously exposed to a familiar and a novel social stimulus. Both Hdc^+/+^ and Hdc^−/−^ mice recognized the familiar juvenile as demonstrated by the significantly higher percentage of time spent exploring the novel compared to the familiar stimulus (Figure 1B; Hdc^+/+^ *p* < 0.01 and Hdc^−/−^ *p* < 0.001). Another set of animals were used to investigate the impact of chronic histamine deprivation on long-term memory (LTM) by performing the retention test 24 h after training (Figure 1A). Under these conditions significant behavioral differences were observed between genotypes. As shown in Figure 1C Hdc^+/+^ mice remembered the previously encountered juvenile, since they spent significantly longer time exploring the novel one (*p* < 0.001). On the contrary, Hdc^−/−^ did not discriminate between the two social stimuli, as revealed by the significant lower discrimination index as compared to Hdc^+/+^ (*p* < 0.01).

**Figure 2:**
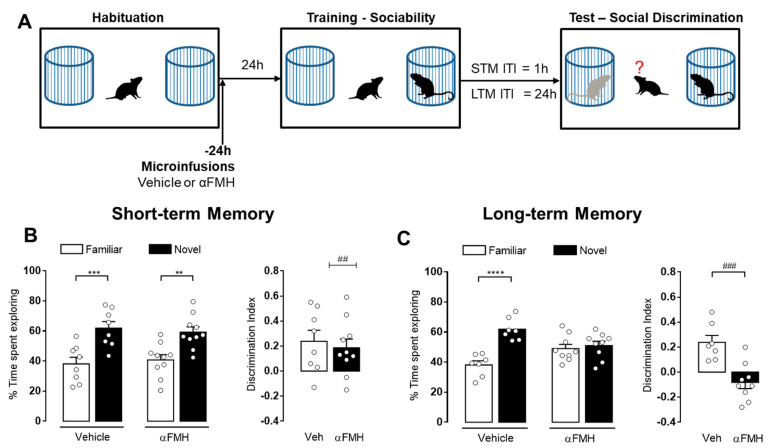
Impact of acute histamine depletion on short- and long-term social recognition memory. (**A**) Schematic drawing showing the sequence of the behavioral procedures. αFMH (5 µg) or vehicle were injected directly into the lateral ventricles of Hdc^+/+^ male mice immediately after habituation. Retention test sessions were performed 1 h (**B**) or 24 h (**C**) after training. Shown are means ± S.E.M. of 7–10 animals per experimental group. **** *p* < 0.0001, *** *p* < 0.001, ** *p* < 0.01 by two-way ANOVA and Bonferroni’s MCT; ^###^ *p* < 0.001, ^##^ *p* < 0.01 by unpaired *t*-test.

The use of knockout mice represents a valuable research tool to investigate the role of specific genes in the unfolding of behaviors, but important limitations associated with this approach exist. For instance, compensatory adjustments due to chronic absence of histamine might contribute to the phenotype observed in Hdc^−/−^ mice. To exclude this mechanism, we evaluated STM and LTM social recognition memory in animals receiving infusions of αFMH, an irreversible inhibitor of histamine biosynthesis, directly into the lateral ventricles immediately after the habituation session (Figure 2A). In previous works we demonstrated that administration αFMH quickly suppressed both baseline and evoked release of histamine. The neurotransmitter levels remained under the sensitivity of the detection method for at least 48 h [36,40,44]. The results obtained are shown in Figure 2. Positive discrimination index values were calculated for both vehicle- and αFMH-infused mice, indicating that despite the treatments received, the animals explored the novel juvenile for a longer time (Figure 2B). Analogous with what was observed for chronically histamine-deprived mice, animals receiving αFMH did not discriminate between the familiar and the novel juvenile when the retention test was performed 24 h after training (*p* < 0.001, Figure 2C).

### 3.3. Inhibition of Histamine Release Impairs Both Short- and Long-Term Social Recognition Memory

**Figure 3:**
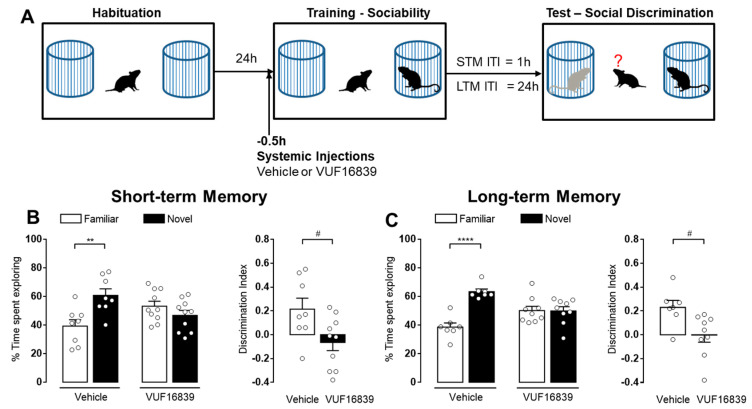
Reduction of histamine release induced by H_3_R activation impairs short- and long-term social recognition memory. (**A**) Schematic drawing showing the sequence of the behavioral procedures. VUF16839 (5 mg/kg, i.p.) or vehicle were injected to Hdc^+/+^ male mice 0.5 h before the training session. Retention tests were performed 1 h (**B**) or 24 h (**C**) after training. Shown are means ± S.E.M. of 7–10 animals per experimental group. **** *p* < 0.0001, ** *p* < 0.01 by two-way ANOVA and Bonferroni’s MCT; ^#^ *p* < 0.05 by unpaired *t*-test.

The endogenous release of histamine in the brain is mainly regulated by the H_3_R autoreceptors [45]. H_3_R activation inhibits, whereas its blockade increases histamine release [46,47]. We therefore, studied the impact of histamine release inhibition on social discrimination. VUF16839, a recently synthesized H_3_R agonist [38] was administered to Hdc^+/+^ male mice at a dose of 5 mg/kg i.p. 30 min before the training session. Controls received vehicle. The retention tests were performed 1 or 24 h later (Figure 3A). In both test sessions, vehicle-treated mice spent longer time exploring the novel social stimulus (*p* < 0.01 STM Figure 3B; *p* < 0.0001 LTM Figure 3C), whereas animals receiving VUF16839 injections did not discriminate between the novel and the familiar juveniles. These findings are further supported by the statistically significant differences observed in the discrimination index between groups (*p* < 0.05 STM Figure 3B, *p* < 0.05 STM Figure 3C). It is important to note that VUF16839 did not alter animals’ sociability (Table 1).

**Figure 4:**
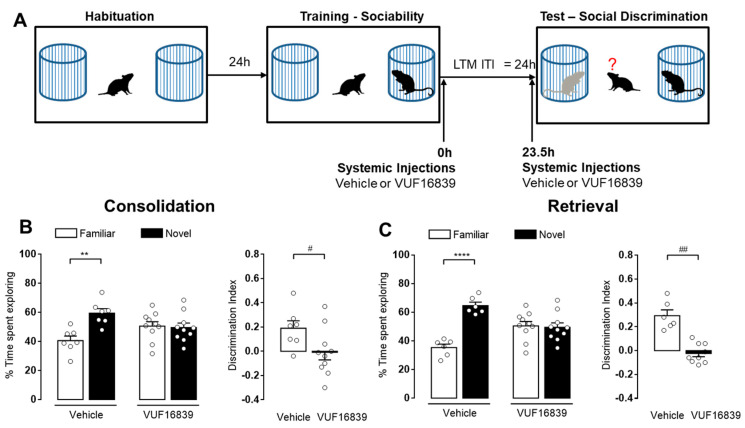
Activation of the H_3_R impairs consolidation and retrieval of long-term social recognition memory. (**A**) Schematic drawing showing the sequence of the behavioral procedures. VUF16839 (5 mg/kg, i.p.) or vehicle were injected to Hdc^+/+^ male immediately (**B**) or 23.5 h after (**C**) training. Retention tests were performed 24 h after training. Shown are means ± S.E.M. of 6–10 animals per experimental group. **** *p* < 0.0001, ** *p* < 0.01 by two-way ANOVA and Bonferroni’s MCT; ^##^ *p* < 0.01, ^#^ *p* < 0.05 by unpaired *t*-test.

The data described above indicate that H_3_R agonism impaired the acquisition of both short- and long-term social recognition memory. Considering that studies evaluating the role of histaminergic transmission on specific memory phases are scant, in the following experiments different groups of Hdc^+/+^ male mice received systemic injections of vehicle or VUF16839 (5 mg/kg) immediately after the training or 30 min before the retention test session, to evaluate the impact of H_3_R activation on long-term social recognition memory consolidation and retrieval, respectively (Figure 4A). As expected, vehicle-treated animals remembered the previous encounter with the familiar juvenile, as they spent a longer time exploring the new one (*p* < 0.01 Figure 4B, *p* < 0.0001 Figure 4C). Conversely, VUF168389-treated mice showed no significant differences in the time spent exploring the social stimuli. The significant differences in the discrimination indexes calculated for vehicle and VUF16839-injected groups further confirmed the impairments in memory consolidation (*p* < 0.05 Figure 4B) and retrieval (*p* < 0.01 Figure 4C).

### 3.4. The H_3_R Agonist-Induced Amnesic Effect Is Prevented by Enhancing Cholinergic Neurotransmission

**Figure 5:**
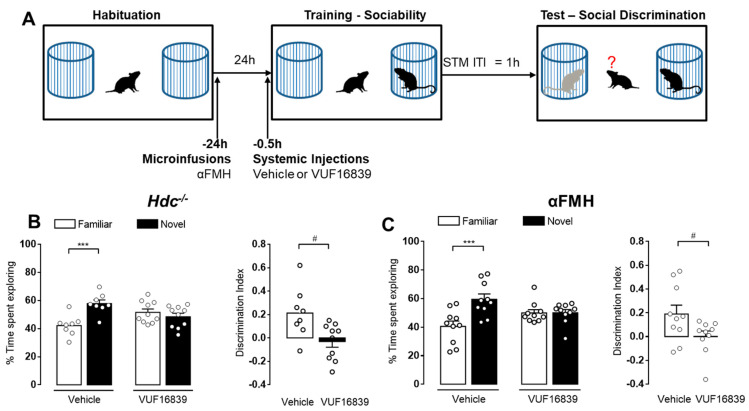
H_3_R agonist-induced amnesic effect does not require histaminergic neurotransmission. (**A**) Schematic drawing showing the sequence of the behavioral procedures. VUF16839 (5 mg/kg, i.p.) or vehicle were injected to Hdc^−/−^ (**B**) or αFMH treated mice (**C**) 0.5 h before training. Retention tests were performed 1 h after training. Shown are means ± S.E.M. of 8–10 animals per experimental group. *** *p* < 0.001 by two-way ANOVA and Bonferroni’s MCT; ^#^ *p* < 0.05 by unpaired *t*-test.

The present study showed that blocking histamine synthesis both genetically or pharmacologically impairs LTM but does not affect STM, whereas H_3_R activation impairs both STM and LTM. Therefore, we hypothesized that VUF1683-induced amnesic effect could be related with its action on H_3_R heteroreceptors decreasing the release of other neurotransmitters [48]. To address this issue, we administered VUF16839 (5 mg/kg, i.p.) to chronically (Hdc^−/−^) or acutely (αFMH i.c.v.) histamine-deprived animals 30 min before training. We then evaluated their performance 1 h later in the social discrimination paradigm (Figure 5A). As described above, both Hdc^−/−^ and αFMH-treated mice receiving vehicle injections showed intact STM being able to discriminate between familiar and novel social stimuli (*p* < 0.001 Figure 5B,C, respectively). When mice received systemic injections of VUF16839, they were no longer able to recognize the previously encountered juvenile, spending similar percentage of time exploring the two social stimuli, which resulted in a statistically significant reduction of the calculated discrimination indexes (*p* < 0.05 Hdc^−/−^ Figure 5A; *p* < 0.05 αFMH Figure 5B). These data indicate that VUF16839-indued amnesia does not require the integrity of the histaminergic system, supporting the involvement of other neurotransmitters.

**Figure 6:**
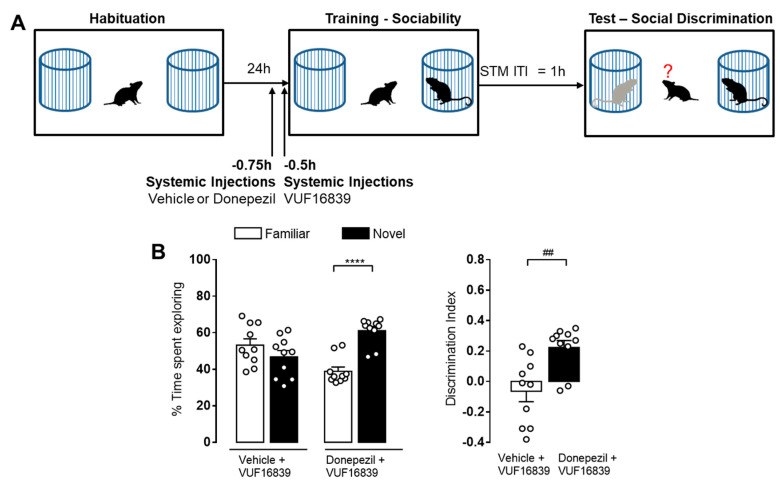
Increased cholinergic transmission prevents H_3_R agonist-induced amnesia. (**A**) Schematic drawing showing the sequence of the behavioral procedures. Different groups of Hdc^+/+^ male mice received donepezil (3 mg/kg, i.p.) or vehicle injections and 15 min latter an i.p. injection of VUF16839 (5 mg/kg). Training session was carried out 30 min after the VUF16839 injection. Retention tests were performed 1 h after training. (**B**) The animals’ performance is shown as means ± S.E.M. (10 animals per experimental group). **** *p* < 0.0001 by two-way ANOVA and Bonferroni’s MCT; ^##^ *p* < 0.001 by unpaired *t*-test.

Given the abundant evidence regarding the interplay between the histaminergic and cholinergic systems on cognition [49], we tested whether increased acetylcholine levels could prevent the cognitive impairment observed following H_3_R activation. Hdc^+/+^ male mice were treated with either vehicle or donepezil (an acetylcholinesterase inhibitor, 3 mg/kg, i.p.) and 15 min later they received a systemic injection of VUF16839 (5 mg/kg, i.p.). Training session was carried out 30 min after the last injection and STM retention was evaluated after 1 h (Figure 6A). As expected, animals treated with vehicle plus VUF16839 did not display significant differences in the percentage of time spent exploring the novel or the familiar stimulus. The mice treated with donepezil in combination with VUF16839, however, spent a significant higher percentage of time exploring the novel juvenile rather than the familiar one (Figure 6B; *p* < 0.0001), resulting in a significant difference in the discrimination index (Figure 6B; *p* < 0.01).

### 3.5. Increased Histamine Release Mediates H_3_R Antagonist-Induced Procognitive Effect

**Figure 7:**
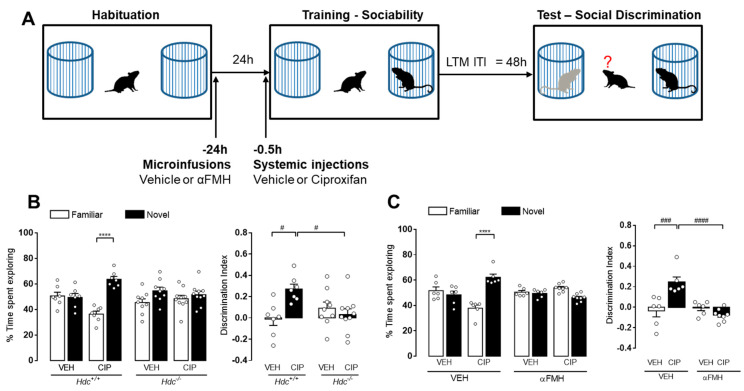
H_3_R antagonist-induced procognitive effect is mediated by histaminergic neurotransmission. (**A**) Schematic drawing showing the sequence of behavioral procedures. Ciproxifan (CIP 3 mg/kg, i.p.) or vehicle were injected to Hdc^−/−^ (**B**) or αFMH infused (**C**) mice 0.5 h before training. Retention tests were performed 48 h after training. Shown are means ± S.E.M. of 6–8 animals by experimental group. **** *p* < 0.0001, ^####^ *p* < 0.0001; ^###^ *p* < 0.001 ^#^ *p* < 0.05 by two-way ANOVA and Bonferroni’s MCT.

The findings described above indicate that reduction of histaminergic neurotransmission invariably impairs long-term recognition memory. Thus, we reasoned that increasing brain histamine availability will have the opposite effect. In order to evaluate this hypothesis, we tested the effects of ciproxifan, a H_3_R antagonist/inverse agonist which blocks H_3_R, hence stimulates histamine release [50]. Hdc^+/+^ male mice received a systemic injection of ciproxifan (3 mg/kg, i.p.) or vehicle 30 min before the training session. In this set of experiments, the retention test was performed 48 h after training (Figure 7A). Vehicle-treated mice displayed no statistically significant difference in the percentage of time spent exploring the familiar or the novel social stimuli (Figure 7B), thus indicating that the passing of time renders memory labile. Mice treated with ciproxifan recognized the previously encountered juvenile, hence they spent more time exploring the new social stimulus (*p* < 0.0001 Figure 7B). As a consequence, the discrimination index was significantly different among experimental groups (*p* < 0.05). These differences cannot be ascribed to changes in animals’ sociability, since ciproxifan treatment did not alter the preference towards the social compartment during training session (Table 1). In order to explore the role of released histamine in mediating such effect, the impact of ciproxifan on social recognition was investigated in histamine-deprived animals (Figure 7A). As shown in Figure 7B, Hdc^−/−^ mice treated with ciproxifan did not distinguish the familiar from the novel social stimulus, as confirmed by the discrimination indices (*p* < 0.05 Figure 7B). We then investigated the effect of ciproxifan on histamine deprived mice injected with αFMH. In analogy to Hdc^−/−^ mice, animals that received αFMH and ciproxifan spent similar percentage of time exploring the familiar and the novel social stimuli (Figure 7C). These results clearly indicate that administration of a selective H_3_R antagonist can potentiate social discrimination memory, but it requires an intact brain histamine system to accomplish this effect in mice.

### 3.6. Histamine Deprivation or Potentiation Do Not Affect General Motor Activity

General motor activity was indirectly accessed by evaluating the total time that the animals spent exploring the two cylinders during the training and retention test phases. As showed in Table 2, no statistically significant differences in the total exploration time emerged across the different experiment, thus suggesting that there are no differences in the motor activity of mice belonging to different genotypes or subjected to pharmacological treatments.

## 4. Discussion

In this study we investigated the participation of the central histaminergic system in mice social behavior separating sociability and the various temporal phases of recognition memory. Our results suggest that brain histamine is not necessary for social preference, while it is essential for the consolidation and retrieval of long-term but for short-term social recognition memory. Furthermore, we dissected the participation of H3 auto- and heteroreceptors in social recognition memory.

Recognition memory refers to the ability of animals and humans to discriminate between familiar and unfamiliar stimuli. Although extensive evidence demonstrates that histamine acting in different brain sites has an important function as a regulator of memory consolidation/retrieval in various learning paradigms [19,26,27], the data regarding histamine-mediated modulation of social discrimination are scarce. Here we combined the use of pharmacological tools (enzymatic inhibitors and specific receptor ligands) and transgenic animals to explore the role of neuronal histamine in rodent’s sociability, short-, and long-term social recognition memory. We evaluated the social discrimination ability of adult mice taking advantage of their innate drive to investigate non-familiar over familiar juvenile conspecifics [7]. This form of recognition is a common process across a variety of mammals, however, there are species and sex differences which influence how it is expressed [1]. For instance, it was observed that recognition memory is much more resilient in mice than it is in rats: the social discrimination ability is reported to last about 30–90 min following acquisition in rat, whereas in mice it was described to last for days [2]. Hence, by adapting the time elapsed between experimental sessions it is possible to dissect the effects on STM and LTM. Our experimental design allowed the investigation of another important component of social behavior, the social preference. Our findings suggest that brain histamine does not have a pivotal role in mice sociability since neither the disruption nor the potentiation of the histaminergic transmission altered the animals’ innate motivation to explore the social stimulus.

In evaluating the animals’ discrimination abilities, we found that neither genetic nor pharmacological disruption of histamine biosynthesis impacted on recognition memory tested 1 h after training (STM), thus suggesting that its formation is independent of histamine neurotransmission. Conversely, when the retention test was performed 24 h after the acquisition (LTM), the integrity of the histaminergic system appeared necessary for the storage of the mnemonic trace. The different effects of silencing the histaminergic system on short- and long-term social recognition memory are in line with reports that lack of histamine impaired LTM, while leaving STM intact in the novel object recognition [37] and the inhibitory avoidance test [44,51]. These findings confirm that STM and LTM are separate processes, as suggested by several reports of drug treatments blocking LTM, while keeping STM intact [52,53,54]. Indeed, the cellular and molecular mechanisms underlying STM stabilization are different from those required for LTM, including a double peak of cAMP-dependent protein kinase activity, accompanied by the phosphorylation of CREB, and both gene expression and protein synthesis [55]. In a previous study we found a good correlation between CREB phosphorylation and the performance of rats in the inhibitory avoidance task (IA). Histamine deprivation by means of αFMH-infusion, impaired LTM while leaving STM intact. It was also found that hippocampal pCREB levels were augmented 10 min and 5 h after IA training in normal rats whereas in the hippocampus of αFMH–treated rats, which displayed long-term memory impairments, an increased CREB phosphorylation was found just 10 min after training, suggesting that histamine activates the CREB pathway to exert its mnemonic effects during the temporal progression of LTM consolidation [44].

Prompted by the finding that brain histamine restriction impairs LTM, but not STM formation, we investigated the effects of the administration of VUF16839, a recently synthesized H_3_R agonist [38] on social discrimination test. H_3_R receptors are localized on histaminergic somata and axons, where they provide negative feedback to restrict histamine synthesis and release [56], and on non-histamine containing neurons where they moderate the release of neurotransmitters such as acetylcholine, dopamine, GABA, glutamate, noradrenaline, and serotonin [46,48]. The administration of VUF16839 before training session impaired not only LTM but also STM, thus showing a different effect on the behavior compared of histamine deprived mice (*Hdc^−/−^* or αFMH treated). LTM deficit elicited by VUF16839 may be the consequence of H_3_ autoreceptors stimulation, that decreases endogenous histamine release. Conversely, VUF16839-induced disruption of STM was histamine-independent, as it occurred also in *Hdc^−/−^* and αFMH-treated mice. The observation that it was antagonized by an acetylcholinesterase inhibitor, donepezil, suggests that an H_3_R-dependent reduction of acetylcholine at the synaptic cleft is a plausible mechanism. Consistently with this view, we reported earlier that systemic administration of H_3_R agonists such as (R)-α-methylhistamine and imetit inhibited cortical acetylcholine release and impaired memory in object recognition, a STM paradigm, in rats [57].

VUF16839 administered immediately after training impaired long-term discrimination, thus suggesting that decreased levels of histamine at the synaptic cleft impaired consolidation of recognition memory. This finding fits well with our previous report that rats failed to consolidate an inhibitory avoidance memory when the brain histaminergic system was silenced [44] and with several studies demonstrating that the histaminergic system modulated memory consolidation in many cognitive tasks such as inhibitory avoidance, fear conditioning and object recognition [37,44,58,59,60]. Taken together, these observations identify a crucial role for the neurotransmitter histamine in this process.

We found that mice injected i.p. with VUF16839 30 min prior to retrieval (therefore almost 24 h after training) showed no significant differences in the time spent exploring the social stimuli. This amnesia is likely caused by an impairment of retrieval rather than consolidation. Indeed, LTM formation, that has been extensively investigated for aversive memories, begins immediately after the training session and progress for little less than 20 h [61,62]. Delayed processes, e.g., phases dependent on gene expression, are involved in LTM maintenance but not its formation [61,63]. Retrieval is not simply a static readout of stored information, rather it represents a dynamic process that requires neuromodulatory signaling similarly to acquisition and consolidation [64]. We reported previously that cerebral histamine depletion impaired retrieval of inhibitory avoidance in rats and blunted retrieval-induced c-Fos activation and cAMP-responsive element binding protein phosphorylation in the CA1 region of the hippocampus [51]. Histamine infusion into the CA1 restored inhibitory avoidance retrieval in histamine-depleted rats by targeting brain histamine H_1_R [51]. We may speculate that long term social recognition shares these features with long term inhibitory avoidance learning, and indicate that VUF16839 by moderating histamine release from the hippocampus causes a reduction of histamine availability at synaptic cleft, thus being responsible for retrieval impairment.

If a deficit of histamine inevitably leads to LTM impairment, its increase would be expected to enhance memory consolidation. To evaluate this hypothesis, we changed the experimental design, by increasing the inter-trial interval to 48 h after training when mice experience physiological forgetting, therefore avoiding the use of injection of amnesic compounds that could interfere with the interpretation of the results. The treatment with the H_3_R antagonist ciproxifan reverted the time-induced recognition deficits, inducing a procognitive effect that relies on histamine neurotransmission. Indeed, it was completely absent in chronically or acutely histamine-depleted animal. H_3_R antagonists enhanced cortical fast rhythms [65,66], that is a feature associated with attention, alertness, and learning. Accordingly, H_3_R antagonists showed positive effects in several preclinical models used to detect procognitive effects [67]. Evidence supporting the involvement of other histamine receptors on social discrimination in mice are scarce. However, it was reported that infusion of ranitidine (an H_2_R antagonist) into the basolateral amygdala and hippocampus harmed the consolidation of social recognition [13]. Therefore, it is conceivable that a two-step action underlies the effects of ciproxifan on memory: antagonism of H_3_R autoreceptors determines an increase of histamine release in the synaptic cleft, which in turn by activating post-synaptic H_2_R facilitates social memory consolidation.

A further contributing factor to the promnesic effect of ciproxifan is the regulation of regional cerebral blood flow (rCBF). A positive relation between resting cerebral blood flow and cognition was recently observed in older adults for global [68,69] and hippocampal cerebral blood flow [70,71]. Histaminergic receptors are expressed in in the endothelium or smooth muscle cells in the cerebral vascular wall [72] and their modulation alters blood-brain barrier permeability and cerebral blood flow [73]. For instance, a dose-dependent increase in hippocampal regional cerebral blood flow (rCBF) was measured following intracerebroventricular infusion of histamine, 2-thiazolylethylamine, and dimaprit, an H_1_R and H_2_R agonist, respectively [74]. Central infusion of clobenpropit, an H3R antagonist which increases brain histamine availability, also significantly increased hippocampal rCBF. Such effect was prevented by infusing mepyramine zolantidine and α-methylhistamine, specific antagonists of the H_1_R, H_2_R, and H_3_R, respectively [75]. Conceivably, also ciproxifan being an H3R antagonist increasing histamine release may increase rCBF contributing to the better cognitive performance observed in this study.

An alternative mechanism could be related with the actions of histamine on glial cells and neuroinflammation. Several studies reported that in physiological conditions histamine induces both astrocytic and microglia reactivity and increase the release of pro-inflammatory cytokines such as TNFα and IL-6 through H_1_R and H_4_R thus sustaining brain inflammation [76,77,78]. However, it was also observed that histamine can counteract lipopolisaccaride (LPS)-induced inflammation, by decreasing microglial migration, phagocytosis, reactive oxygen species production, as well as the release of IL-1β and prostaglandin E2 [79,80]. Significant progress in establishing the effects of specific cytokines in the brain on learning, memory and plasticity has been done. However, most of the studies have also uncovered contradictory roles of cytokines in modulation of memory [81]. A modern theory hypothesizes that in physiological conditions the pro-inflammatory cytokines such as IL-1β, IL-6, and TNFα support long-term plasticity and learning and memory processes. On the contrary, when cytokine levels are elevated, such as in models of brain injury or infection or neurodegeneration, the effects of cytokines are mostly detrimental to memory function [82]. In this regard, in APP/PS1 transgenic mice, a model of Alzheimer’s disease, chronic treatment with ciproxifan reduced both COX-1 and COX-2 activities, decreased the level of pro-inflammatory cytokines IL-1α, IL-1β, and IL-6, and increased the level of anti-inflammatory cytokine TGF-1β [83]. Due to the relevant differences between this and our study (acute *versus* chronic treatment, normal *versus* transgenic animals) we believe that the ciproxifan-induced modulation of cytokine release probably had a minor impact in the observed results.

## 5. Conclusions

The present study suggests that (i) brain histamine is not involved in mice sociability; (ii) the integrity of the histaminergic system in the brain is crucial for recognition long-term, but not short-term, memory formation; (iii) the integrity of the brain histaminergic system is also required for recognition LTM retrieval; (iv) ciproxifan, an H_3_R antagonist, showed a procognitive effect in recognition LTM. Advances in the understanding of histaminergic mechanisms underlying memory processes may help in the search for cognitive disorder. Here, we provide evidence that targeting the histaminergic system may modify the encoding, consolidation, and retrieval of certain types memory.

## Figures and Tables

**Table 1:** Effects of different manipulations on animals’ behavior during the training session.

	Genotype	Treatments		Time Spent Exploring (%)		*p*	Sociability Index (SI)		
		i.c.v.	i.p.	Social	Non Social				
Figure 1B	*Hdc^+/+^*	-	-	71.6 ± 11.3	28.4 ± 11.3	****	0.43 ± 0.23	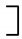	ns
	*Hdc^−/−^*	-	-	68.6 ± 9.5	31.4 ± 9.5	****	0.37 ± 0.19		
Figure 1C	*Hdc^+/+^*	-	-	73.3 ± 5.9	27.7 ± 5.9	****	0.44 ± 0.12	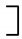	ns
	*Hdc^−/−^*	-	-	68.6 ± 4.84	31.4 ± 4.8	***	0.37 ± 0.10		
Figure 2B	*Hdc^+/+^*	Veh	-	70.4 ± 3.4	29.6 ± 3.4	****	0.41 ± 0.19	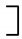	ns
	*Hdc^+/+^*	αFMH	-	76.29 ± 3.2	23.71 ± 3.2	****	0.52 ± 0.20		
Figure 2C	*Hdc^+/+^*	Veh	-	83.5 ± 7.9	16.5 ± 7.9	****	0.52 ± 0.16	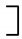	ns
	*Hdc^+/+^*	αFMH	-	72.3 ± 10.7	27.7 ± 10.7	****	0.64 ± 0.19		
Figure 3B	*Hdc^+/+^*	-	Veh	71.1 ± 15.7	28.9 ± 15.7	****	0.42 ± 0.31	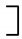	ns
	*Hdc^+/+^*	-	VUF16839	81.9 ± 7.2	18.1 ± 7.2	****	0.64 ± 0.14		
Figure 3C	*Hdc^+/+^*	-	Veh	67.8 ± 6.9	32.2 ± 6.9	****	0.35 ± 0.14	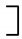	ns
	*Hdc^+/+^*	-	VUF16839	62.5 ± 9.5	37.5 ± 9.5	****	0.25 ± 0.19		
Figure 4B	*Hdc^+/+^*	-	Veh	72.3 ± 5.9	27.7 ± 5.9	****	0.45 ± 0.12	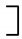	ns
	*Hdc^+/+^*	-	VUF16839	65.7 ± 8.8	34.3 ± 8.8	****	0.31 ± 0.18		
Figure 4C	*Hdc^+/+^*	-	Veh	71.8 ± 6.5	28.2 ± 6.5	****	0.44 ± 0.13	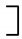	ns
	*Hdc^+/+^*	-	VUF16839	65.6 ± 7.9	34.4 ± 7.9	****	0.31 ± 0.16		
Figure 5B	*Hdc^−/−^*	-	Veh	67.0 ± 7.4	33.0 ± 7.4	****	0.34 ± 0.15	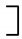	ns
	*Hdc^−/−^*	-	VUF16839	79.7 ± 7.6	30.3 ± 7.6	****	0.39 ± 0.15		
Figure 5C	*Hdc^+/+^*	αFMH	Veh	70.4 ± 9.9	29.6 ± 9.9	****	0.41 ± 0.20	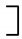	ns
	*Hdc^+/+^*	αFMH	VUF16839	66.9 ± 12.4	33.1 ± 12.4	****	0.34 ± 0.25		
Figure 6	*Hdc^+/+^*	-	Veh + VUF16939	79.3 ± 10.8	20.7 ± 10.8	****	0.58 ± 0.21	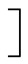	ns
	*Hdc^+/+^*	-	Donepezil + VUF16839	68.7 ± 18.3	31.3 ± 18.3	****	0.38 ± 0.37		
Figure 7B	*Hdc^+/+^*	-	Veh	71.2 ± 6.5	28.7 ± 6.5	****	0.42 ± 0.13	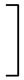	ns
	*Hdc^+/+^*	-	Ciproxifan	66.6 ± 5.4	33.4 ± 5.4	****	0.33 ± 0.11		
	*Hdc^+/+^*	-	Veh	67.1 ± 6.2	32.9 ± 6.2	****	0.34 ± 0.12		
	*Hdc^+/+^*	-	Ciproxifan	74.6 ± 5.7	25.4 ± 5.7	****	0.48 ± 0.11		
Figure 7C	*Hdc^+/+^*	Veh	Veh	64.5 ± 5.1	35.5 ± 5.1	****	0.29 ± 0.25	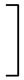	ns
	*Hdc^+/+^*	αFMH	Ciproxifan	71.2 ± 2.3	28.8 ± 2.3	****	0.42 ± 0.12		
	*Hdc^+/+^*	Veh	Veh	69.5 ± 2.6	30.5 ± 2.6	****	0.39 ± 0.13		
	*Hdc^+/+^*	αFMH	Ciproxifan	70.7 ± 3.1	29.3 ± 3.1	****	0.41 ± 0.17		

Social preference is assessed by the difference in the percentage of time spent exploring the cup containing a conspecific juvenile (social) versus the empty cup (non-social). Sociability index (SI) was calculated as described in Material and Methods. All the results are reported as mean ± SD of 6–10 animals per experimental group (*** *p* < 0.001; **** *p* < 0.0001 by Two Way ANOVA and Bonferroni’s MCT).

**Table 2:** Effects of different manipulations on mice exploratory activity. The results are reported as mean ± SD of the total time the animals spent exploring the cups during training and retention test sessions. *n* = 6–10 animals by experimental group.

	Genotype	Treatments		Time Spent Exploring (s)					
		i.c.v.	i.p.	Training	*p*		Test	*p*	
Figure 1B	*Hdc^+/+^*	-	-	180.5 ± 46.5	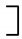	ns	122.1 ± 52.6	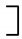	ns
	*Hdc^−/−^*	-	-	161.9 ± 22.0			124.2 ± 24.9		
Figure 1C	*Hdc^+/+^*	-	-	177.9 ± 39.9	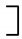	ns	178.1 ± 64.7	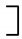	ns
	*Hdc^−/−^*	-	-	142.5 ± 42.0			150.6 ± 28.9		
Figure 2B	*Hdc^+/+^*	Veh	-	116.3 ± 40.1	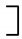	ns	128.8 ± 43.8	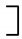	ns
	*Hdc^+/+^*	αFMH	-	95.8 ± 21.1			138.9 ± 45.5		
Figure 2C	*Hdc^+/+^*	Veh	-	146.5 ± 46.2	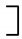	ns	178.1 ± 64.7	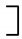	ns
	*Hdc^+/+^*	αFMH	-	114.9 ± 47.1			127.9 ± 61.6		
Figure 3B	*Hdc^+/+^*	-	Veh	113.6 ± 53.6	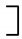	ns	115.1 ± 35.8	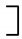	ns
	*Hdc^+/+^*	-	VUF16839	163.6 ± 46.9			151.2 ± 73.0		
Figure 3C	*Hdc^+/+^*	-	Veh	138.4 ± 59.6	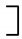	ns	144.0 ± 66.7	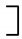	ns
	*Hdc^+/+^*	-	VUF16839	110.3 ± 35.4			100.0 ± 43.1		
Figure 4B	*Hdc^+/+^*	-	Veh	126.1 ± 53.5	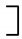	ns	150.1 ± 57.3	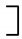	ns
	*Hdc^+/+^*	-	VUF16839	113.3 ± 32.7			108.7 ± 22.5		
Figure 4C	*Hdc^+/+^*	-	Veh	163.7 ± 60.9	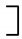	ns	140.6 ± 72.4	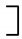	ns
	*Hdc^+/+^*	-	VUF16839	139.8 ± 62.5			102.6 ± 42.6		
Figure 5B	*Hdc^−/−^*	-	Veh	156.3 ± 70.4	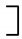	ns	143.8 ± 31.5	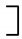	ns
	*Hdc^−/−^*	-	VUF16839	107.8 ± 40.8			112.2 ± 42.6		
Figure 5C	*Hdc^+/+^*	αFMH	Veh	100.8 ± 35.4	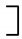	ns	124.9 ± 61.6	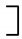	ns
	*Hdc^+/+^*	αFMH	VUF16839	86.9 ± 25.5			88.5 ± 33.2		
Figure 6	*Hdc^+/+^*	-	Veh + VUF16939	79.5 ± 23.4	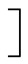	ns	82.2 ± 31.3	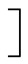	ns
	*Hdc^+/+^*	-	Donepezil + VUF16839	72.7 ± 26.6			101.4 ± 17.4		
Figure 7B	*Hdc^+/+^*	-	Veh	99.7 ± 33.5	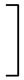	ns	115.0 ± 26.5	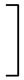	ns
	*Hdc^+/+^*	-	Ciproxifan	138.8 ± 33.2			95.6 ± 20.6		
	*Hdc^+/+^*	-	Veh	112.7 ± 29.9			93.3 ± 42.3		
	*Hdc^+/+^*	-	Ciproxifan	119.7 ± 27.2			101.5 ± 36.2		
Figure 7C	*Hdc^+/+^*	Veh	Veh	110.3 ± 19.3	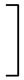	ns	104.2 ± 27.5	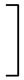	ns
	*Hdc^+/+^*	αFMH	Ciproxifan	145.7 ± 35.3			106.8 ± 31.9		
	*Hdc^+/+^*	Veh	Veh	134.7 ± 47.1			134.8 ± 45.7		
	*Hdc^+/+^*	αFMH	Ciproxifan	118.2 ± 19.1			135.9 ± 30.1		

## Data Availability

The data presented in this study are available on request from the corresponding author.

## Supplementary Materials

The following are available online at https://www.mdpi.com/article/10.3390/biom11040555/s1, Tables S1–S4: Statistical Analysis. Table S1: Critical values obtained in the statistical analysis of the raw data regarding the time spent exploring the cups during the training session (Table 1), Table S2: Critical values obtained from the statistical analysis of the raw data regarding the Sociability index (Table 1), Table S3: Critical values obtained in the statistical analysis of the raw data regarding the time spent exploring the cups containing the social stimuli in the retention test session (Figure 1, Figure 2, Figure 3, Figure 4, Figure 5, Figure 6 and Figure 7), Table S4: Critical values obtained from the statistical analysis of the raw data regarding the Discrimination index (Figure 1, Figure 2, Figure 3, Figure 4, Figure 5, Figure 6 and Figure 7).
