# Supplementary material for: Short- and Long-Term Social Recognition Memory Are Differentially Modulated by Neuronal Histamine
Source: Biomolecules. 2021 Apr 9;11(4):555. doi: 10.3390/biom11040555 (PMC8069616; doi:10.3390/biom11040555)
Supplement: Supplementary file 1 [file biomolecules-11-00555-s001.pdf]

Supplementary Material related to the manuscript:

Short- and long-term social recognition memory are differentially modulated by neuronal histamine

Barbara Rani, Bruna Silva-Marques, Rob Leus, Maria Beatrice Passani, Patrizio Blandina and Gustavo Provensi

This PDF file includes:

Table S1: Critical values obtained in the statistical analysis of the raw data regarding the time spent exploring the cups during the training session (Table 1)

Table S2: Critical values obtained from the statistical analysis of the raw data regarding the Sociability index (Table 1)

Table S3: Critical values obtained in the statistical analysis of the raw data regarding the time spent exploring the cups containing the social stimuli in the retention test session (Figures 1-7)

Table S4: Critical values obtained from the statistical analysis of the raw data regarding the Discrimination index (Figures 1-7)

Table S1: Critical values obtained in the statistical analysis of the raw data regarding the time spent exploring the cups during the training session (Table 1)

Figure	n	Analysis (post hoc reported in Table 1)	Factors Analyzed		F ratios	P values
**1B**	6	two-way ANOVA (Bonferroni)	Genotype *(Hdc$^{+/+}$ vs Hdc$^{-/-}$)* Cups (Non social *vs* Social)	Interaction	F (1, 20) = 0,4975	P = 0,4887
				Genotype	F (1, 20) = 2,088e-014	P > 0,9999
				Cups	F (1, 20) = 89,18	P < 0,0001
**1C**	6-7	two-way ANOVA (Bonferroni)	Genotype *(Hdc$^{+/+}$ vs Hdc$^{-/-}$)* Cups (Non social *vs* Social)	Interaction	F (1, 22) = 2,964	P = 0,0992
				Genotype	F (1, 22) = 4,493e-013	P > 0,9999
				Cups	F (1, 22) = 365,1	P < 0,0001
**2B**	8-10	two-way ANOVA (Bonferroni)	Treatments (α-FMH *vs* Vehicle) Cups (Non social *vs* Social)	Interaction	F (1, 32) = 3,234	P = 0,0816
				Treatments	F (1, 32) = 0,0	P > 0,9999
				Cups	F (1, 32) = 199,9	P < 0,0001
**2C**	7-9	two-way ANOVA (Bonferroni)	Treatments (α-FMH *vs* Vehicle) Cups (Non social *vs* Social)	Interaction	F (1, 28) = 10,79	P < 0,01
				Treatments	F (1, 28) = 1,484e-014	P > 0,9999
				Cups	F (1, 28) = 267,3	P < 0,0001
**3B**	8-10	two-way ANOVA (Bonferroni)	Treatments (VUF16839 *vs* Vehicle) Cups (Non social *vs* Social)	Interaction	F (1, 32) = 7,617	P < 0,01
				Treatments	F (1, 32) = 0,0	P > 0,9999
				Cups	F (1, 32) = 180,4	P < 0,0001
**3C**	7-9	two-way ANOVA (Bonferroni)	Treatments (VUF16839 *vs* Vehicle) Cups (Non social *vs* Social)	Interaction	F (1, 28) = 2,965	P = 0,0961
				Treatments	F (1, 28) = 8,807e-014	P > 0,9999
				Cups	F (1, 28) = 100,0	P < 0,0001
**4B**	7-10	two-way ANOVA (Bonferroni)	Treatments (VUF16839 *vs* Vehicle) Cups (Non social *vs* Social)	Interaction	F (1, 30) = 5,947	P < 0,05
				Treatments	F (1, 30) = 1,690e-013	P > 0,9999
				Cups	F (1, 30) = 196,1	P < 0,0001
**4C**	6-8	two-way ANOVA (Bonferroni)	Treatments (VUF16839 *vs* Vehicle) Cups (Non social *vs* Social)	Interaction	F (1, 26) = 5,024	P < 0,05
				Treatments	F (1, 26) = 3,676e-013	P > 0,9999
				Cups	F (1, 26) = 183,0	P < 0,0001
**5B**	8-10	two-way ANOVA (Bonferroni)	Treatments (VUF16839 *vs* Vehicle) Cups (Non social *vs* Social)	Interaction	F (1, 32) = 1,192	P = 0,2830
				Genotype	F (1, 32) = 6,855e-014	P > 0,9999
				Cups	F (1, 32) = 212,8	P < 0,0001
**5C**	10	two-way ANOVA (Bonferroni)	Treatments (VUF16839 *vs* Vehicle) Cups (Non social *vs* Social)	Interaction	F (1, 36) = 0,9587	P = 0,3340
				Genotype	F (1, 36) = 0,0	P > 0,9999
				Cups	F (1, 36) = 110,0	P < 0,0001

**6B**	10	two-way ANOVA (Bonferroni	Treatments (Donepezil+Vuf16839 *vs* Vehicle+VUF16839) Cups (Non social *vs* Social)	Interaction	F (1, 36) = 8,891	P < 0,01
				Genotype	F (1, 36) = 9,346e-015	P > 0,9999
				Cups	F (1, 36) = 131,5	P < 0,0001
**7B**	7-10	two-way ANOVA (Bonferroni	Genotype (*Hdc*$^{+/+}$ *vs Hdc*$^{-/-}$) /Treatments (Ciproxifan *vs* Vehicle) Cups (Non social *vs* Social)	Interaction	F (3, 58) = 6,379	P < 0,001
				Groups	F (3, 58) = -1,461e-013	P > 0,9999
				Cups	F (1, 58) = 714,3	P < 0,0001
**7C**	6-8	two-way ANOVA (Bonferroni	Injection (*α*-FMH *vs* Vehicle) /Treatments (Ciproxifan *vs* Vehicle) Cups (Non social *vs* Social)	Interaction	F (3, 44) = 1,498	P = 0,2282
				Groups	F (3, 44) = 3,535e-014	P > 0,9999
				Cups	F (1, 44) = 238,5	P < 0,0001

Table S2: Critical values obtained from the statistical analysis of the raw data regarding the Sociability index (Table 1)

Figure	"n"	Analysis (post hoc reported in table 1)	t	P values
**1B**	6	t-Test	0.4988	P = 0,6287
**1C**	6-7	t-Test	1.237	P = 0,2417
**2B**	8-10	t-Test	1.286	P = 0,2166
**2C**	7-9	t-Test	1.371	P = 0,1920
**3B**	8-10	t-Test	2.002	P = 0,0625
**3C**	7-9	t-Test	1.195	P = 0,2520
**4B**	7-10	t-Test	1.724	P = 0,1052
**4C**	6-8	t-Test	1.593	P = 0,1352
**5B**	8-10	t-Test	0.7684	P = 0,4535
**5C**	10	t-Test	0.6774	P = 0,5068
**6B**	10	t-Test	1.883	P = 0,0759
**7B**	7-10	One-way ANOVA (Bonferroni)	F (3, 29) = 2,574	P = 0,0732
**7C**	6-8	One-way ANOVA (Bonferroni)	F (3, 22) = 0,7486	P = 0,5348

Table S3: Critical values obtained in the statistical analysis of the raw data regarding the time spent exploring the cups containing the social stimuli in the retention test session (Figures 1-7)

Figure	n	Analysis (post hoc reported in figures)	Factors Analyzed	F ratios		P values
**1B**	6	two-way ANOVA (Bonferroni)	Genotype *($Hdc^{+/+}$ vs $Hdc^{-/-}$)* Stimuli (Familiar *vs* Novel)	Interaction	F (1, 20) = 0,5395	P = 0,4712
				Genotype	F (1, 20) = 4,035e-014	P > 0,9999
				Stimuli	F (1, 20) = 33,16	P < 0,0001
**1C**	6-7	two-way ANOVA (Bonferroni)	Genotype *($Hdc^{+/+}$ vs $Hdc^{-/-}$)* Stimuli (Familiar *vs* Novel)	Interaction	F (1, 22) = 12,18	P = 0,0021
				Genotype	F (1, 22) = -6,488e-015	P > 0,9999
				Stimuli	F (1, 22) = 6,335	P < 0,01
**2B**	8-10	two-way ANOVA (Bonferroni)	Treatments (α-FMH *vs* Vehicle) Stimuli (Familiar *vs* Novel)	Interaction	F (1, 32) = 0,4536	P = 0,5055
				Treatments	F (1, 32) = 1,348e-014	P > 0,9999
				Cups	F (1, 32) = 29,43	P < 0,0001
**2C**	7-9	two-way ANOVA (Bonferroni)	Treatments (α-FMH *vs* Vehicle) Stimuli (Familiar *vs* Novel)	Interaction	F (1, 28) = 19,88	P = 0,0001
				Treatments	F (1, 28) = 9,711e-014	P > 0,9999
				Stimuli	F (1, 28) = 14,17	P < 0,001
**3B**	8-10	two-way ANOVA (Bonferroni)	Treatments (VUF16839 *vs* Vehicle) Stimuli (Familiar *vs* Novel)	Interaction	F (1, 32) = 12,42	P < 0,01
				Treatments	F (1, 32) = 0,0	P > 0,9999
				Stimuli	F (1, 32) = 3,630	P = 0,0658
**3C**	7-9	two-way ANOVA (Bonferroni)	Treatments (VUF16839 *vs* Vehicle) Stimuli (Familiar *vs* Novel)	Interaction	F (1, 28) = 19,33	P < 0.001
				Treatments	F (1, 28) = 0,1120	P = 0,7404
				Stimuli	F (1, 28) = 18,60	P < 0,001
**4B**	7-10	two-way ANOVA (Bonferroni)	Treatments (VUF16839 *vs* Vehicle) Stimuli (Familiar *vs* Novel)	Interaction	F (1, 30) = 9,508	P < 0,01
				Treatments	F (1, 30) = -4,259e-014	P > 0,9999
				Stimuli	F (1, 30) = 7,725	P < 0,01
**4C**	6-8	two-way ANOVA (Bonferroni)	Treatments (VUF16839 *vs* Vehicle) Stimuli (Familiar *vs* Novel)	Interaction	F (1, 26) = 61,25	P < 0,0001
				Treatments	F (1, 26) = 1,104e-013	P > 0,9999
				Stimuli	F (1, 26) = 63,62	P < 0,0001
**5B**	8-10	two-way ANOVA (Bonferroni)	Treatments (VUF16839 *vs* Vehicle) Stimuli (Familiar *vs* Novel)	Interaction	F (1, 32) = 13,76	P < 0,001
				Genotype	F (1, 32) = 8,032e-015	P > 0,9999
				Cups	F (1, 32) = 6,287	P < 0,05
**5C**	10	two-way ANOVA (Bonferroni)	Treatments (VUF16839 *vs* Vehicle) Stimuli (Familiar *vs* Novel)	Interaction	F (1, 36) = 9,248	P < 0,01
				Genotype	F (1, 36) = 0,0	P > 0,9999
				Stimuli	F (1, 36) = 9,405	P < 0,01

**6B**	10	two-way ANOVA (Bonferroni	Treatments (Donepezil+VUF16839 *vs* Vehicle+VUF16839)<br>Stimuli (Familiar *vs* Novel)	Interaction	F (1, 36) = 23,58	P < 0,0001
				Genotype	F (1, 36) = 0,0	P > 0,9999
				Stimuli	F (1, 36) = 7,162	P < 0,05
**7B**	7-10	two-way ANOVA (Bonferroni	Genotype *($Hdc^{+/+}$ vs $Hdc^{-/-}$)* + Treatments (Ciproxifan *vs* Vehicle)<br>Stimuli (Familiar *vs* Novel)	Interaction	F (3, 56) = 10,05	P < 0,0001
				Groups	F (3, 56) = 4,784e-014	P > 0,9999
				Stimuli	F (1, 56) = 20,66	P < 0,0001
**7C**	6-8	two-way ANOVA (Bonferroni	Injection *(α-FMH vs* Vehicle*)* + Treatments (Ciproxifan *vs* Vehicle)<br>Stimuli (Familiar *vs* Novel)	Interaction	F (3, 44) = 25,32	P < 0,0001
				Groups	F (3, 44) = 5,769e-014	P > 0,9999
				Stimuli	F (1, 44) = 4,571	P < 0,05

Table S4: Critical values obtained from the statistical analysis of the raw data regarding the Discrimination index (Figures 1-7)

Figure	n	Analysis (post hoc reported in figures)	t	P values
**1B**	6	t-Test	0.6345	P = 0,5400
**1C**	6-7	t-Test	2.492	P < 0.05
**2B**	8-10	t-Test	0.4786	P = 0,6387
**2C**	7-9	t-Test	4.299	P < 0,001
**3B**	8-10	t-Test	2.480	P < 0,05
**3C**	7-9	t-Test	2.732	P < 0,05
**4B**	7-10	t-Test	2.166	P < 0,05
**4C**	6-8	t-Test	5.512	P < 0,001
**5B**	8-10	t-Test	2.777	P < 0,001
**5C**	10	t-Test	2.153	P < 0,05
**6B**	10	t-Test	3.454	P < 0,01
**7B**	7-10	One-way ANOVA (Bonferroni)	F (3, 29) = 4,438	P < 0,05
**7C**	6-8	One-way ANOVA (Bonferroni)	F (3, 22) = 12,82	P < 0,0001
